# Agonism and Antagonism at the Insulin Receptor

**DOI:** 10.1371/journal.pone.0051972

**Published:** 2012-12-27

**Authors:** Louise Knudsen, Bo Falck Hansen, Pia Jensen, Thomas Åskov Pedersen, Kirsten Vestergaard, Lauge Schäffer, Blagoy Blagoev, Martin B. Oleksiewicz, Vladislav V. Kiselyov, Pierre De Meyts

**Affiliations:** 1 Receptor Systems Biology Laboratory, Hagedorn Research Institute, Gentofte, Denmark; 2 Department of Insulin and Incretin Biology, Hagedorn Research Institute, Gentofte, Denmark; 3 Insulin Biology, Novo Nordisk A/S, Måløv, Denmark; 4 Biochemistry and Molecular Biology, University of Southern Denmark, Odense M, Denmark; 5 Arsanis Biosciences, Vienna, Austria; University of Cambridge, United Kingdom

## Abstract

Insulin can trigger metabolic as well as mitogenic effects, the latter being pharmaceutically undesirable. An understanding of the structure/function relationships between insulin receptor (IR) binding and mitogenic/metabolic signalling would greatly facilitate the preclinical development of new insulin analogues. The occurrence of ligand agonism and antagonism is well described for G protein-coupled receptors (GPCRs) and other receptors but in general, with the exception of antibodies, not for receptor tyrosine kinases (RTKs). In the case of the IR, no natural ligand or insulin analogue has been shown to exhibit antagonistic properties, with the exception of a crosslinked insulin dimer (B29-B’29). However, synthetic monomeric or dimeric peptides targeting sites 1 or 2 of the IR were shown to be either agonists or antagonists. We found here that the S961 peptide, previously described to be an IR antagonist, exhibited partial agonistic effects in the 1–10 nM range, showing altogether a bell-shaped dose-response curve. Intriguingly, the agonistic effects of S961 were seen only on mitogenic endpoints (^3^H-thymidine incorporation), and not on metabolic endpoints (^14^C-glucose incorporation in adipocytes and muscle cells). The agonistic effects of S961 were observed in 3 independent cell lines, with complete concordance between mitogenicity (^3^H-thymidine incorporation) and phosphorylation of the IR and Akt. Together with the B29-B’29 crosslinked dimer, S961 is a rare example of a mixed agonist/antagonist for the human IR. A plausible mechanistic explanation based on the bivalent crosslinking model of IR activation is proposed.

## Introduction

The insulin receptor (IR) is a member of the receptor tyrosine kinase (RTK) family [1]–[6], which includes the receptors for insulin, insulin-like growth factors (IGFs) and many other growth factors. The RTKs consist of an extracellular portion containing the ligand binding sites, a transmembrane helix, and an intracellular portion with tyrosine kinase activity. Ligand binding triggers activation of the tyrosine kinase activity, involving autophosphorylation of tyrosines around the catalytic site [7]. The extracellular domain of the IR exists under two alternatively spliced forms, IR-A and IR-B, depending on the absence or presence, respectively, of a 12 amino acid segment encoded by exon 11 [3], [4]. The intracellular portion of the IR contains seven tyrosine phosphorylation sites, two in the juxtamembrane domain (JM), Y965 and Y972, three in the tyrosine kinase (TK) domain, Y1158, Y1162, and Y1163, and the last two in the carboxy-terminal tail, Y1328 and Y1334 (IR-B numbering).

The binding of insulin to the IR is described by a curvilinear Scatchard plot, which suggests the existence of high- and low-affinity binding sites and/or negative cooperativity [8]. Furthermore, dissociation of prebound labelled insulin from the IR is accelerated by an excess of non-labelled insulin in comparison to dissociation in buffer alone, a hallmark of negative cooperativity [9]. At supraphysiological concentrations of non-labelled insulin (above 100 nM), the accelerated dissociation of labelled insulin is abolished due to self-antagonism. Models describing these complex binding interactions between insulin and the IR were proposed in 1994 by Schäffer [10] and De Meyts [8]. Both models assume that each IR half contains two binding sites, sites 1 and 2. The insulin molecule crosslinks the two IR halves by binding to site 1 on one α-subunit and site 2 on the other α-subunit, thereby creating a high-affinity interaction, leaving the other two IR sites for interaction with insulin with a lower affinity. In order to explain the acceleration of dissociation of prebound labelled insulin by unlabelled insulin (negative cooperativity), De Meyts [8] proposed that IR sites 1 and 2 are disposed in an antiparallel symmetry, allowing alternative crosslinking of the two pairs of binding sites. In 2006 the crystal structure of the ectodomain dimer of IR was solved [11] and confirmed the antiparallel arrangement of the binding sites. A 5-parameter mathematical model for this complex interaction was recently developed by Kiselyov et al. [12] based on the concept of a harmonic oscillator, which was able to reproduce the essential kinetic features of the ligand-receptor interaction and to provide robust estimates of the parameters (site rate constants and crosslinking constant). Recently, by using the model, the differences in insulin binding kinetics between the two IR isoforms were determined allowing accurate determination of the binding kinetics of the individual sites as well as the apparent affinities [13].

Interestingly, despite the apparent complexity and multi-subsite nature of the binding interaction, all natural ligands of the IR (animal insulins) as well as dozens of chemically modified or genetically engineered insulin analogues over the past four decades were always found to have full agonistic properties with widely divergent potencies in metabolic bioassays like rodent adipocytes lipogenesis (same maximum with dose-response curves shifting left or right). The only exception was a covalent insulin dimer crosslinked between the two B29 lysines, which showed both antagonistic and partial agonistic properties [14]. The mitogenic properties of the IR (e.g. in ^3^H-thymidine incorporation assays) have not been as thoroughly investigated for possible antagonism, again with the exception of the crosslinked dimer which antagonized mitogenesis [14].

In 2002, peptides binding to the IR binding sites were generated by phage display [15] in order to define the molecular architecture of the receptor and to identify the critical regions (“hotspots”) required for biological activity in a site-directed manner. Two groups of phage-derived peptides were found to bind to or close to the two insulin-binding sites. A third group of phage-derived peptides did not compete for binding to insulin sites 1 and 2, and were therefore named site 3 peptides. Surprisingly, some of the site 1 peptides stimulated glucose uptake in adipocytes with partial or full agonistic activity, even though they were presumably not able to crosslink the IR. In contrast, site 2 and 3 peptides acted as glucose uptake antagonists. In terms of IR phosphorylation, site 1 peptides acted as either agonists or antagonists, whereas site 2 and site 3 peptides acted only as antagonists. Finally, site 1 peptides also bound to the IGF-IR, in contrast to site 2 and 3 peptides, which bound exclusively to the IR [15].

Several combinations of homo-and heterodimers of site 1 and 2 peptides were generated in order to increase the affinity for the IR and to achieve a more insulin-like activation mechanism of the IR [16]. Interestingly, heterodimers of site 1 and 2 peptides acted as either agonists or antagonists, depending on the order of peptide linkage. Heterodimers comprising a site 1 peptide C-terminally linked to the N-terminal end of a site 2 peptide acted as antagonists (these heterodimers are termed site 1–2 peptides). In contrast, heterodimers comprising a site 2 peptide C-terminally linked to the N-terminal end of a site 1 peptide acted as agonists (these heterodimers are termed site 2–1 peptides) [16]. However, Jensen et al. [17] recently found that a site 2–1 peptide named S597 was a full agonist on glycogen synthesis (with a decreased potency), but a weak inducer of cell proliferation in rat L6 myoblast cells overexpressing the human IR-A. Interestingly, the authors found that S597 was able to antagonize the effect of insulin on cell proliferation down to the effect of S597 alone, indicating that S597 is not a full but a partial agonist for mitogenesis [17]. This prompted us to examine more closely the properties of the site 1–2 peptide S961, nearly identical to S661 [18] reported to be a full IR antagonist, and investigate whether it may also have agonistic properties on the IR.

## Experimental Procedures

### Insulin, IGF-I and S961

Human insulin (S100, aqueous solution), S661 and S961 were provided by Novo Nordisk A/S, Denmark. The sequence of S961 is identical to the sequence of S661 shown in Schäffer et al. 2008 [18] except that S961 is a C-terminal acid, whereas S661 is a C-terminal amide. The two peptides have been shown to have identical properties [18]. S961 was dissolved in DMSO for the mitogenicity assays and in H_2_O for the IR and Akt activation assays. S661 was dissolved in DMSO. IGF-I (Sigma; I-3769) was dissolved in 10 mM HCl.

### Cell Culture

L6 rat myoblasts (L6-WT) (ATCC; CRL-1458), and L6 rat myoblasts overexpressing the A isoform of the human IR (L6-hIR) [19] were cultured at 37°C in a 5% CO_2_ humidified atmosphere in Nunc culture flasks. The growth medium was DMEM with 5 mM glucose (Gibco; 21885-025) supplemented with 10% v/v fetal bovine serum (Gibco; 26140-079) and 1% v/v penicillin/streptomycin/glutamine (Gibco; 10378-016). For L6-hIR cells, the medium was supplemented with 1 mg/ml G418. Cells were subcultured 3 times per week, with a split ratio of 1∶10 for two days and 1∶20 for three days. L6-hIR cells used for the IR/AKT phosphorylation assays were cultured as explained before except that the growth medium was DMEM with 5 mM glucose (Gibco; 21885-025) supplemented with 10% v/v fetal bovine serum (Gibco; 16140-0711), 1% v/v penicillin/streptomycin (Gibco; 15140-122), and 1 mg/ml G418 (Gibco; 10131-019).

MCF-7 human mammary adenocarcinoma cells (ATCC; HTB-22) [20], [21] were cultured at 37°C in a 5% CO_2_ humidified atmosphere in Nunc culture flasks. Growth medium was D-MEM with 25 mM glucose with phenol red (Lonza; Bio Whittaker BE12-61F) supplemented with 10% v/v fetal bovine serum (Gibco; 26140-079), 1% v/v penicillin/streptomycin/glutamine (Gibco; 10378-016), 1% v/v non-essential amino acids (MEM 100X) (Gibco; 11140-035) and 1.5 µM recombinant human insulin (Ballerup Apotek; 013950). Cells were subcultured 2 times weekly, with a split ratio of 1∶4.

3T3-L1 mouse fibroblast-like preadipocytes (ATCC; CL-173) [22] were cultured at 37°C in a 10% CO_2_ humidified atmosphere in Nunc culture dishes. Growth medium was D-MEM with 25 mM glucose (Gibco; 41966-029) supplemented with 10% v/v donor calf serum (Gibco; 16030-041), 1% v/v penicillin/streptomycin (Cambrex; DE17-602E), and 1% v/v L-Glutamine (Lonza; BE17-605E). Cells were subcultured every second day with a split ratio of 1∶5.

For differentiation, 3T3-L1 cells were allowed to reach 100% confluence, followed by further incubation for 48 hours in fresh medium. Then, differentiation into adipocytes was initiated by incubating the cells for 48 hours in differentiation medium: DMEM, 10% v/v fetal bovine serum, 1% v/v penicillin/streptomycin, 1% v/v L-Glutamine, 115 µg/ml 1-methyl-3-isobutylxanthine, 390 ng/ml dexamethasone and 1 µg/ml insulin. After 2 days, the cells were incubated for another 48 hours in differentiation medium without 1-methyl-3-isobutylxanthine and dexamethasone. Then, the cells were incubated for another 48 hours in differentiation medium without 1-methyl-3-isobutylxanthine, dexamethasone and insulin. Finally, the medium was renewed (differentiation medium without 1-methyl-3-isobutylxanthine, dexamethasone and insulin) and the cells were used for glycogen synthesis experiments after 1–2 days.

Chinese hamster ovary cells overexpressing the A isoform of the human IR (CHO-hIR) [23] were cultured at 37°C in a 5% CO_2_ humidified atmosphere in Nunc culture flasks. Growth medium was DMEM (Gibco; 21885-025) with 5.5 mM glucose (1000 mg/l) supplemented with 10% v/v fetal bovine serum, 1% v/v penicillin/streptomycin (Gibco; 11140), 100 µM MEM non essential amino acids (Gibco; 11140) and 100 µM methotrexate (Wyeth Lederle; 062661). Cells were subcultured 2 times weekly with at split ratio of 1∶25 for three days and 1∶50 for four days.

### Mitogenicity Assays with Human Insulin and IGF-I in L6-WT, L6-hIR and MCF-7 Cells

For all three cell lines, genomic DNA synthesis was quantified by incorporation of ^3^H-thymidine (Amersham Biosciences).

The L6-WT and L6-hIR mitogenicity assays were performed essentially as previously described [19]. Briefly, cells were synchronized by a combination of topoinhibition and serum starvation, and stimulated for 18–19 hours in medium containing 0.1% serum supplemented with increasing concentrations of ligand. Then, cells were pulse-labelled with 0.125 µCi/well ^3^H-thymidine for 2 hours, and incorporated ^3^H-thymidine was quantitated by scintillation counting.

The MCF-7 mitogenicity assay was performed essentially as previously described [20], [21]. Briefly, cells were synchronized by a combination of topoinhibition and serum starvation, and stimulated for 24 hours Dulbecco’s medium without phenol red (Gibco; 11880-028) containing 0.1% serum and 1% v/v penicillin/streptomycin/glutamine supplemented with increasing concentrations of ligand. Then, cells were pulse-labelled with 0.125 µCi/well ^3^H-thymidine for 2 hours, and incorporated ^3^H-thymidine was quantitated by scintillation counting.

### Mitogenicity Assays in the Presence of S961

For all cell lines, two hours prior to insulin or IGF-I stimulation, the medium was replaced with starvation medium containing S961 at the concentrations indicated in figures and results. The cells were incubated at 37°C in a 5% CO_2_ humidified atmosphere for 2 hours, at which time HI or IGF-I were added and mitogenicity assays were carried out as described above. Thus, insulin and IGF-I stimulation occurred in the continued presence of S961. Negative-control cultures received an equivalent volume of DMSO instead of S961.

### Glycogen Synthesis Assay in Differentiated 3T3-L1 Adipocytes

Differentiated 3T3-L1 cells seeded in 12-well plates were starved for three hours in 1 ml/well starvation medium consisting of DMEM with 1 g/l glucose (Lonza; BE12-707F) supplemented with 1% v/v penicillin/streptomycin and 1% v/v L-Glutamine. Next, 10 µl of insulin, IGF-I, or S961 were added, followed by 20 µl ^14^C-uridine diphosphoglucose (UDPG, PerkinElmer NEC042), and the cells were incubated for 1 hour at 37°C. The cells were washed three times with 1 ml ice cold PBS pH 7.4 (Gibco; 14080-048), and lysed in 1 ml ice cold 1N NaOH for 30 minutes on ice. The cell lysates were transferred to Sarstedt tubes (catalogue number 60.540.012), and heated for 30 minutes in a water bath at 90°C. Next, 285 µl 4 mg/ml glycogen (Sigma; G-0885) was added, followed by vortexing, addition of 2 ml ice cold 96% ethanol, and incubation overnight at -20°C, for glycogen to precipitate. Precipitated glycogen was collected by centrifugation at 4°C for 20 minutes at 2800 rpm in a Heraeus Megafuge 1.0R centrifuge. Supernatants were discarded, the precipitated glycogen washed with 2 ml 96% ethanol followed by centrifugation, and the pellets dried by turning tubes upside down for a few minutes. The precipitated glycogen was dissolved in 525 µl H_2_O, of which 500 µl were mixed with 10 ml OptiPhase Hisafe 3 scintillation fluid (Perkin Elmer; 1200-437), followed by counting for 600 seconds in a Beta counter. Positive and negative controls consisted of 1 µl UDPG in 10 ml OptiPhase Hisafe 3, and 10 ml Optiphase Hisafe 3 alone, respectively.

### Glycogen Synthesis Assay in L6-hIR Cells

L6-hIR cells were seeded in Cytostar plates (Perkin Elmer; RPNQ0163) with a density of 40,000 cells/well. Growth medium was DMEM with 5 mM glucose (Gibco; 21885-025) supplemented with 10% v/v FBS (Gibco; 16140-071), 1% v/v penicillin/streptomycin (Gibco; 15140-122) and 1 mg/ml G418 (Gibco; 10031-019). After 24 hours, the medium was exchanged with 200 µl starvation medium consisting of MEM alpha MEM (Gibco; 041-94645) supplemented with 20 mM Hepes (Gibco; 15630-056), 2 mM Glutamin (Gibco 25030-024), 2 mM Pyruvate (Gibco; 11360-039), 1% v/v penicillin/streptomycin, 0.1% Human Serum Albumin (Sigma; A-1887) and 500 µM glucose. The cells were starved for 3 hours followed by 24 hours of stimulation with increasing concentrations of ligand and 10µl ^14^C- Deoxy-D-Glucose (Perkin Elmer; NEC 495A001MC), the latter diluted 1∶10 in starvation medium. The plates were sealed with backing tape (Perkin Elmer; 6005199) and radioactivity was measured in a TopCounter (Perkin Elmer).

### Lipogenesis in Primary Rat Adipocytes

Primary rat adipocytes were prepared from epididymal fat-pads excised from 110–140 g Sprague Dawley rats as previously described [23], [24]. Briefly, fine minced tissue was shaken at 1 fat-pad per 1.5 ml of degradation buffer consisting of 110 mM NaCl (Merck 106404), 4.8 mM KCl (Merck 104936), 1.2 mM KH_2_PO_4_ (Merck 104873), 1.2 mM MgSO_4_ (Merck 105886), 12.6 mM CaCl_2_ (Merck 102382), 0.4 mg/ml collagenase (Worthinton Biochemical Corporation), 4% HSA (Sigma-Aldrich A1887), 1 mM glucose (Sigma G-7528), and 25 mM Hepes (Sigma; H9136) in polypropylene vials between 1–1½ hours at 36.5°C at 190 rpm.

Next, the suspension was filtered through a syringe with two layers of gaze and washed twice in 20 ml wash buffer consisting of 20% v/v Krebs stock buffer, 25 mM Hepes and 1% v/v HSA, where after the liquid phase was aspirated. Following lipocrit measurement, the adipocytes were resuspended to reach a final concentration of 0.5% cells. 110 µl of the resuspended cells with ^3^H-glucose (Perkin Elmer; NET331A001MC, 1 mCi/ml) were incubated with 10 µl insulin or insulin analogue dilutions in a final concentration of 0.5 mM glucose. Final concentration of ^3^H-glucose was 0.125 µCi/well were from Perkin Elmer. For each ligand eight different doses were used. The dilutions were made in wash buffer. Adipocytes were incubated at 36.5°C in a Labtherm incubator shaking at 120 minutes^-1^. The reaction was terminated by adding 100 µl Microscient E (PerkineElmer) and ^3^H-radioactivity was measured in a TopCounter NXT (Perkin Elmer) counter for 2 minutes/well.

### IR and Akt Phosphorylation Assays

CHO-hIR cells were seeded in 12-well plates at a density of 250,000 cells/well, grown for 48 hours at 37°C in growth medium, and shifted to assay medium consisting of DMEM (Gibco; 21885-025) with 5.5. mM glucose, 1% v/v penicillin/streptomycin and 0.1% human serum albumin (Sigma; A-1887). Cells were stimulated with increasing concentrations of ligand for 30 minutes at 37°C. Reactions were stopped by washing monolayers three times in 1 ml ice-cold PBS (Gibco; 14080-048), followed by snap freezing in liquid nitrogen and lysis in 100 µl lysis buffer (Bio Source; FNN0011) containing 1 mM AEBSF (Calbiochem; 101500) and protease inhibitor cocktail (Sigma; P-2714). Protein concentration in lysates was determined using the Pierce BCA method. IR and Akt phosphorylation was quantitated by sandwich ELISA, as recommended by the manufacturer (Bio Source/Invitrogen). Briefly, diluted lysates were applied to a 96-well plates containing immobilized monoclonal antibodies specific for either human/mouse/rat IR (β-subunit) or human/mouse/rat Akt, and incubated for 2 hours at room temperature. The antibodies were part of an ELISA kit which has the following catalog numbers; IR(pY972) cat no. KHR9141, IR(pY1158) cat no. KHR9121, IR(pYpY1162/1163) cat no. KHR9131, IR(pY1328) cat no. KHR9151, IR(pY1334) cat no. KHR9161 and AKT(pS473) cat no. KHO0111. Following washing, phosphorylation-specific rabbit antibodies were added, followed by horseradish peroxidase-labelled anti-rabbit IgG and development with chromogen (TMB).

The procedure for the L6-hIR cells was essentially as described for the CHO-hIR cells except that the cells were plated with a cell density of 125,000 cells/well and incubated for three days before the previously described growth medium for L6-hIR was shifted into assay medium for the IR/AKT phosphorylation assays.

## Results

### S961 Stimulated a Mitogenic Response in L6-hIR Cells

**Figure 1:**
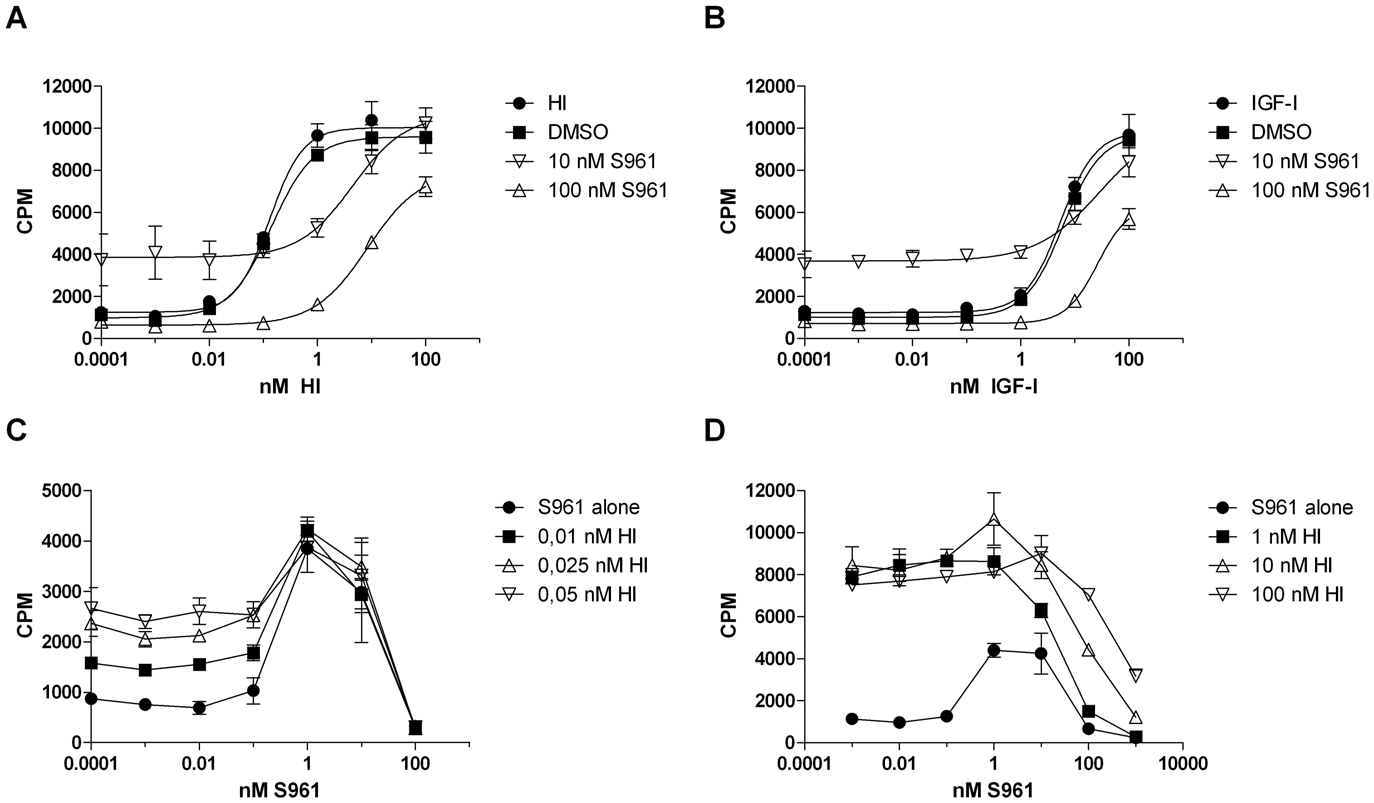
S961 has antagonist as well as agonist activity on IR-mediated mitogenic effect in L6-hIR cells. A, "10 nM S961" and "100 nM S961" curves: Cells were pretreated for 2h with 10 nM or 100 nM S961, and stimulated with increasing concentrations of insulin (as indicated on the x-axis) in the continued presence of S961. "HI" curve, insulin stimulation only (without S961). "DMSO" curve, insulin stimulation with equal volume DMSO added instead of S961. **B,** "10 nM S961" and "100 nM S961" curves: Cells were pretreated for 2h with 10 nM or 100 nM S961, and stimulated with increasing concentrations of IGF-I (as indicated on the x-axis) in the continued presence of S961. "IGF-I" curve, IGF-I stimulation only (without S961). "DMSO" curve, IGF-I stimulation with equal volume DMSO added instead of S961. **C,** "0.01 nM HI", "0.025 nM HI" and "0.05 nM HI" curves: Cells were pretreated for 2 h with increasing concentrations of S961 (as indicated on the x-axis), and stimulated with 0.01 nM, 0.025 nM or 0.05 nM HI in the continued presence of S961. "S961 alone" curve, insulin was omitted. **D**, "1 nM HI", "10 nM HI" and "100 nM HI" curves: Cells were pretreated for 2 h with increasing concentrations of S961 (as indicated on the x-axis), and stimulated with 1 nM, 10 nM or 100 nM HI in the continued presence of S961. "S961 alone" curve, insulin was omitted. **A** and **B,** Graphs are representative for three independent experiments, each experiment comprising triplicate determinations of each ligand concentration. **C,** The graph is performed in triplicates once. **D,** The graph is representative for two independent experiments each performed in triplicates. Error bars indicate one standard deviation.

Usually, in mammalian cells, IGF-I is a stronger mitogen than insulin [20], [21]. However, in L6-hIR cells, insulin and IGF-I had mitogenic potencies (EC50 values) of 0.13 nM and 5.41 nM, respectively (Fig. 1). In this regard, L6-hIR cells are unusually responsive to the mitogenic effect of human insulin. This was in agreement with a previous report [19], supporting that in L6-hIR cells, the mitogenic effect of insulin is primarily mediated by the transfected human IR.

First, because the initial assumption was that S961 is a pure antagonist [18] we performed L6-hIR cell proliferation assays where cells were pre-treated for 2h with 10 nM or 100 nM S961, followed by insulin or IGF-I stimulation in the continued presence of S961. Negative controls consisted of insulin and IGF-I stimulated cells that received an equivalent volume of DMSO instead of S961. At S961 concentrations of 100 nM, the mitogenic potency of human insulin was reduced 100-fold (Fig. 1A), and the mitogenic potency of human IGF-I was reduced 10-fold (Fig. 1B), as shown by the rightward shift of the dose-response curves. In the absence of S961, insulin at below 10 pM and IGF-I at below 1 nM did not stimulate mitogenic responses in L6-hIR cells, as expected (Fig. 1A and 1B). Surprisingly, in the presence of S961 at 10 nM, cell proliferation was observed even at insulin levels below 10 pM and IGF-I levels below 1 nM (Fig. 1A and 1B). Both for insulin in the 0.1 - 10 pM range, and IGF-I in the 0.1 pM - 1 nM range, the increased cell proliferation at 10 nM S961 compared to 100 nM S961 was highly statistically significant (Fig. 1A and 1B, P<0.0005, two-tailed t-test). These results suggested that S961 had not only antagonistic but also agonistic properties.

In order to verify the agonistic effects of S961, we performed a dose-response curve with S961 alone in L6-hIR cells. At concentration of 1 nM, S961 significantly enhanced cell proliferation in comparison to 0.01 nM, (Fig. 1C, P<0.005, two-tailed t-test), whereas the increase in cell proliferation at 10 nM S961 was not statistically significant (Fig. 1C, P = 0.055, two-tailed t-test). At 100 nM S961, the mitogenic effect disappeared (Fig. 1C, "S961 alone" curve). Together, these findings supported that S961 was a mixed agonist/antagonist, with antagonist effects dominant above 10 nM, and agonist activities dominant in the 1–10 nM range, resulting in a bell-shaped curve.

We then examined the effect of low concentrations of insulin on S961-treated cells. The insulin concentrations chosen for this were 0.01 nM, 0.025 nM and 0.05 nM, just at and slightly above the threshold concentration where insulin started to stimulate a mitogenic response in L6-hIR cells (Fig. 1A, "HI" curve). At S961 concentrations of 1 and 10 nM, which corresponded to the maximal agonist activity of S961, the three insulin concentrations did not further increase ^3^H-thymidine incorporation (Fig. 1C, compare all curves at the 1 and 10 nM x-axis point). In contrast, at S961 concentrations below 1 nM, the low insulin concentrations stimulated an additive mitogenic response (Fig. 1C, compare all curves in the 0.001–0.1 nM x-axis range. P<0.05, two-tailed t-test). This supported that S961 does not exhibit antagonistic activity below 1 nM.

Finally, we examined maximal and supramaximal insulin concentrations corresponding to the maximal mitogenic effect of insulin in S961-pretreated cells (Fig. 1D). This experiment confirmed that above 10 nM, S961 is a strong IR antagonist. Approximately 10-fold molar excess of S961 was needed to neutralize the mitogenic effect of insulin in L6-hIR cells (Fig. 1D).

In summary, all mitogenicity results from L6-hIR cells were concordant, supporting that S961 was a mixed agonist/antagonist, with antagonistic effects dominating above 10 nM and agonistic effects dominating in the 1–10 nM range.

### S961 Stimulated a Mitogenic Response in MCF-7 Cells

**Figure 2:**
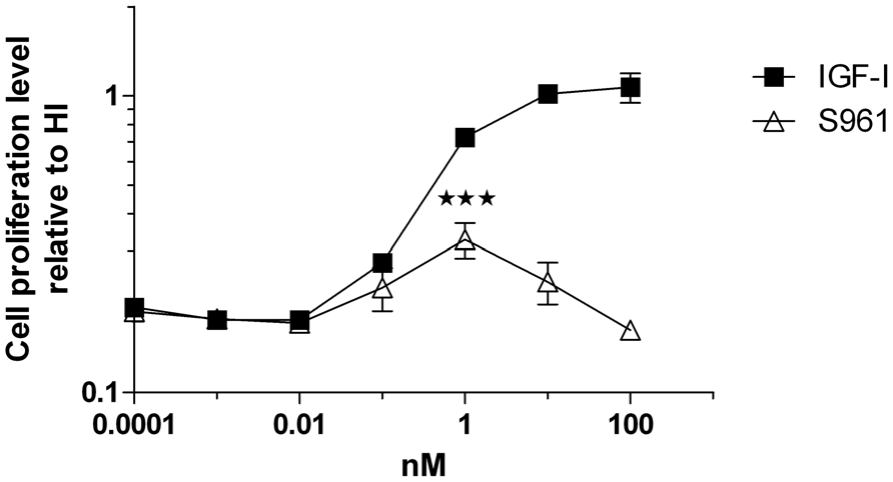
Agonistic (mitogenic) effect of S961 in MCF-7 cells. Cells were stimulated with increasing concentrations of S961 or IGF-I. The graph is representative for three experiments. The increased response for S961 at 1 nM compared to the response at the three lowest concentrations is statistically significant (P<0.001, two-tailed t-test). Data points represent means of triplicate determinations. Error bars show one standard deviation.

In order to examine the dose dependant S961 effects on mitogenicity in cancer cells expressing endogenous IR and IGF-IR we performed ^3^H-thymidine incorporation in MCF-7 cells with S961 and IGF-I. S961 at 1 nM but not at higher concentrations significantly increased cell proliferation in MCF-7 cells (Fig. 2), although to a lesser degree than in L6-hIR cells, showing that the agonistic effect of S961 was not an artefact of the L6-hIR cell system.

### S961 Stimulated IR and Akt Phosphorylation in CHO-hIR Cells

**Figure 3:**
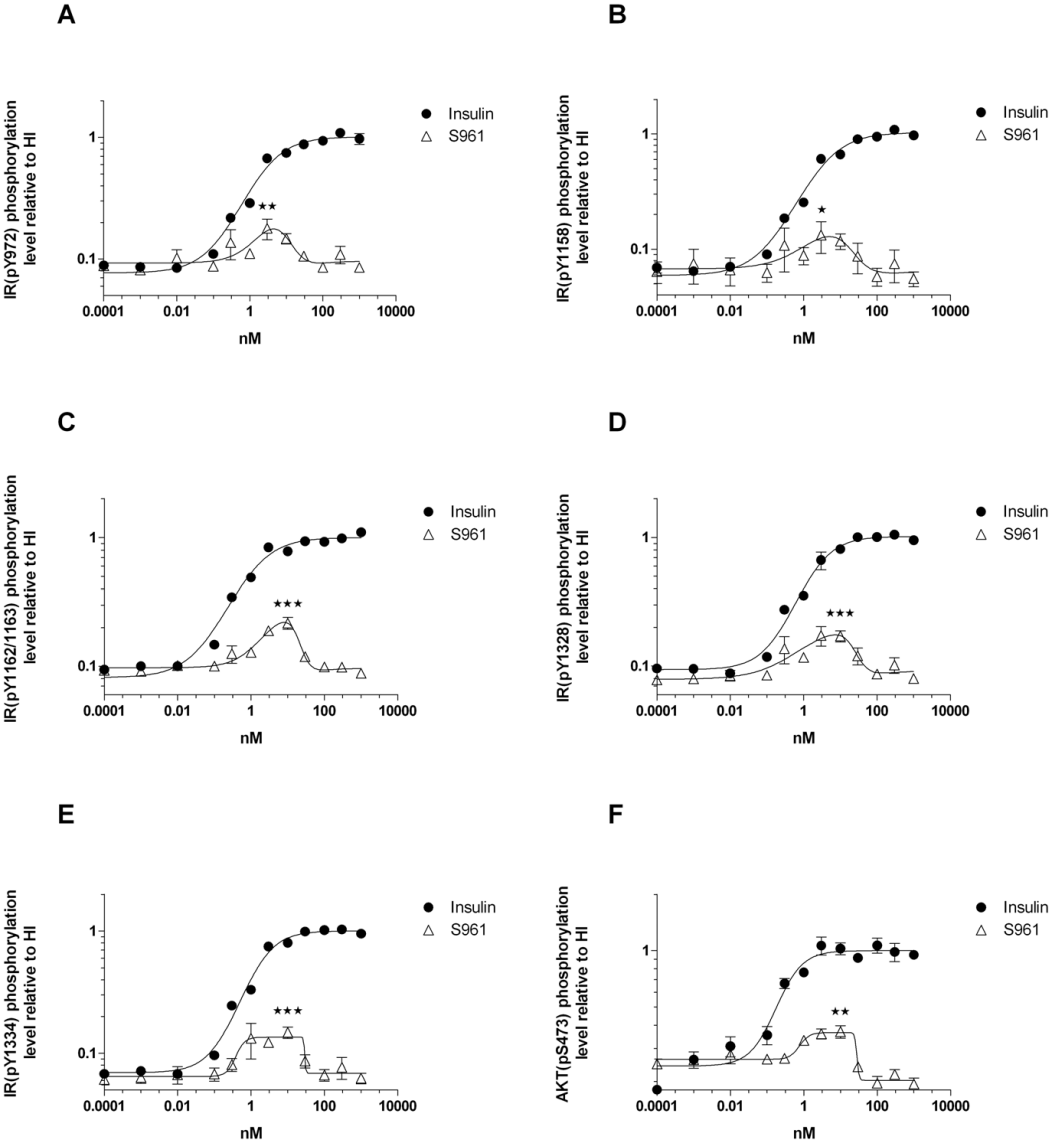
S961 stimulates IR and Akt phosphorylation in CHO-hIR cells. Cells were stimulated with increasing concentrations of HI or S961. **A**-**E**, IR tyrosine phosphorylation. The 6 tyrosine phosphorylation sites which were examined were Y972 in the juxtamembrane domain, Y1158 and Y1162/1163 in the tyrosine kinase domain, and Y1328 and Y1334 in the C-terminal tail end of the IR. **F**, Akt phosphorylation. Phosphorylation of Ser437 is known to be required for Akt activation. Panels **A**-**E**: the increased tyrosine phosphorylation of the IR was significant (compared to 0.0001 nM, 0.001 nM and 0.01 nM S961, P<0.05*, P<0.01**, P<0.001***, two-tailed t-test). Panel **F**: the increased serine phosphorylation of Akt was significant (compared to 0.0001 nM, 0.001 nM and 0.01 nM S961, P<0.01**, two-tailed t-test). Data points represent average of three independent experiments, each comprising triplicate determinations. Error bars show one standard deviation.

**Figure 4:**
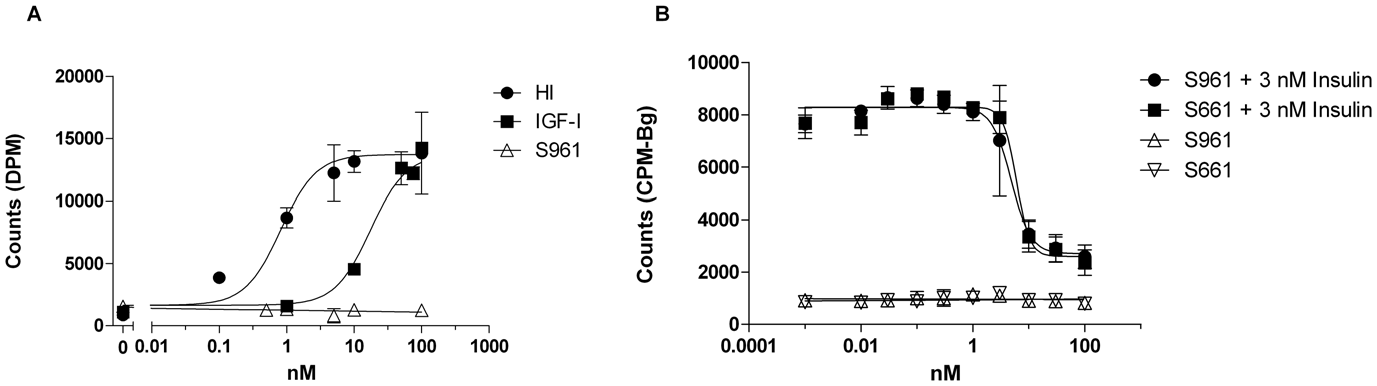
S961 did not stimulate glycogen synthesis in differentiated adipocytes or in muscle cells. A , Differentiated 3T3-L1 adipocytes were stimulated with increasing concentrations of HI, IGF-I or S961. The graph is representative of two independent experiments each comprising duplicate determinations. Error bars show one standard deviation. **B**, L6-hIR muscle cells were stimulated with increasing concentrations of S961/S661 alone or in combination with 3 nM insulin. The graph is representative of two independent experiments each comprising triplicate determinations. Error bars show one standard deviation.

We showed that S661, which has been previously reported to perform in a similar way as S961 [18], behaved as an antagonist with respect to IR and AKT phosphorylation (Fig. S1), thus confirming the antagonistic properties of the peptide. S961 concentrations of 1 and 10 nM significantly stimulated tyrosine phosphorylation of the IR (Fig. 3A–E), including the three sites in the tyrosine kinase domain critical for IR activation (Fig. 3B and 3C), i.e. Y1158 and Y1162/1163 in the TK domain, as well as Y1328 and Y1334 in the C-terminal tail end of the IR, and Y972 in the JM domain. Furthermore, S961 concentrations of 1 and 10 nM significantly stimulated Akt phosphorylation at serine 473, known to be critical for the activation of Akt [25] (Fig. 4F).

The S961 dose-response curves for IR and Akt phosphorylation in CHO-hIR cells and the dose-response curves for mitogenicity in L6-hIR and MCF-7 cells coincided perfectly, with maximum at 1 and 10 nM peptide (compare Fig. 1C and 1D with Fig. 2 and Fig. 3A–E).

### S961 did not Stimulate Glycogen Synthesis in Differentiated Adipocytes or in Muscle Cells

We investigated if S961 was able to stimulate other biological endpoints than cell proliferation. We therefore performed glycogen synthesis assays with HI, IGF-I and S961 in differentiated 3T3-L1 adipocytes (Fig. 4A) and with S961 alone or in combination with HI in L6-hIR cells (Fig. 4B). As expected, HI and IGF-I were strong and very weak stimulators, respectively, of glycogen synthesis in differentiated 3T3-L1 cells in contrast to S961 which did not induce glycogen synthesis in differentiated adipocytes (Fig. 4A). Similarly, neither S961 nor S661 were able to stimulate glycogen synthesis in L6-hIR cells (Fig. 4B). In addition, both S961 and S661 antagonized the effect of 3 nM insulin with identical potency (Fig. 4B). S661 was included in this experiment to verify peptides similarity.

### S961 did not Induce Lipogenesis in Adipocytes

**Figure 5:**
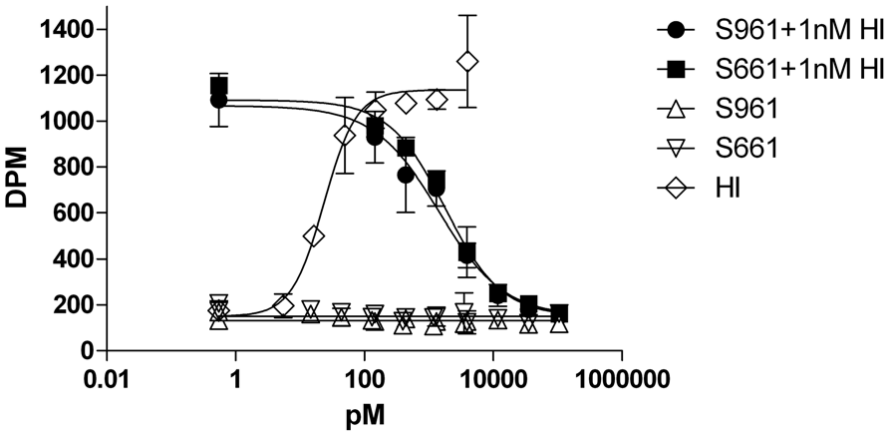
S961 did not stimulate lipogenesis in rat adiopocytes. Primary rat adipocytes were stimulated with increasing concentrations of S961or S661 alone or in combination with 1 nM insulin. Insulin alone was included as a reference. The graph is representative of two independent experiments each comprising duplicate determinations. Error bars show one standard deviation.

To rule out the possibility that S961 was able to initiate other metabolic pathways than glycogen synthesis, we performed lipogenesis in rat adipocytes. Consistent with the results from glycogen synthesis, S961 and S661, in contrast with insulin, were not able to initiate an agonistic response, but were fully capable of antagonizing the effect of 1 nM insulin (Fig. 5).

## Discussion

Agonism and antagonism at orthosteric or allosteric sites are pharmacological properties of receptors that are well described for the GPCRs [26] and growth hormone/cytokine classes of receptors [27]. Self-antagonism in the latter class of receptors has also been described, resulting in bell-shaped dose-response curves [27], [28]. In the case of RTKs, various strategies to design agonists or antagonists are possible, as described in ref. [29]. Small molecules aimed at inhibiting the TK domain (tyrphostins) have been described for the EGF and other growth factor receptors [30]. Monoclonal antibodies with antagonistic properties have been used successfully to target the ErbB2 receptor, and have made it to the clinic as anti-cancer therapies [31].

In the case of the IR, no natural ligand (various animal insulins) or modified ligand (analogues) has ever been found to be antagonistic in metabolic assays (such as lipogenesis in isolated rodent fat cells) despite the study of dozens of modified insulins. The sigmoid dose-response curves exhibit variable potencies (with leftward or rightward shift relative to insulin) but with the same maximal response. A natural mutant insulin (Leu B24 insulin) was initially claimed to be an antagonist *in vitro* [32], [33] but was soon demonstrated by others not to be an antagonist either *in vitro* [34]–[36] or *in vivo* [37]. A notable exception is a covalent insulin dimer crosslinked between the two B29 lysines, which showed antagonistic and partial agonistic properties in both metabolic and mitogenic assays [14]. The only property of the IR for which antagonism with several insulin analogues has been demonstrated is the negative cooperativity [8]. Dose-response-curves for acceleration of dissociation of pre-bound labelled insulin by unlabelled insulin in an infinite dilution is bell-shaped [8], [9], indicating self-antagonism. Some insulin analogues modified at the C-terminal end of the B-chain (“cooperative site”) [38] or at the N-terminal end of the A-chain (Aladdin H. and De Meyts, P. unpublished data) do not induce the accelerated tracer dissociation and antagonize the accelerating effect of native insulin [8]. These features are readily explainable in the framework of the harmonic oscillator model of the IR [12]. A variety of monoclonal antibodies for the IR and IGF-IR have been shown, depending on their binding epitopes, to be either agonists, neutral or antagonists [39]–[42]. More recently, some monomeric and dimeric peptides targetting IR site 1 and site 2 (described in the introduction) were shown to behave as antagonists of biological effects of insulin *in vitro* and *in vivo* [16], [18].

We have investigated here more closely the properties of the site 1–2 dimeric peptide S961, similar to S661 that was previously described as an antagonist [18]. Using three different cell lines (L6-hIR, MCF-7 and CHO-hIR), we showed that S961 is in fact a mixed agonist/antagonist on mitogenic signalling from the IR and that S961 has agonistic effects on IR phosphorylation and Akt phosphorylation endpoints. In all 3 cell lines, S961 exhibited agonistic activity between 1 and 10 nM. The results from all 3 cell culture systems were highly consistent. Thus, the mixed agonist/antagonist properties of S961 were unlikely to be a cell culture artefact. Intriguingly, the agonist activity of S961 was observed only with mitogenicity and IR/Akt phosphorylation endpoints. On the glucose incorporation endpoint in differentiated 3T3-L1 preadipocytes, in L6-hIR cells and in rat adipocytes S961 had no agonistic effects. In addition, we found that S661 behaved in the same manner as S961 with respect to lipogenesis and glycogen synthesis.

Based on the EC50 values of HI and IGF-I, the mitogenic effect of insulin in L6-hIR cells can be reasonably assumed to be mediated by the transfected human IR-A. Additionally, S961 has been reported to be highly IR-specific, with a selectivity for the IR versus the IGF-IR that is higher than that of insulin itself (the IGF-IR affinity of S961 in comparison to HI is 3%, and the IR-A affinity of S961 in comparison to HI is 60% [18]). In addition, a contribution from IR/IGF-IR hybrids [43] is likely since S961, unlike insulin, binds to hybrid receptors with high affinity [18]. In MCF-7 cells, the agonistic effect of S961 is likely induced through IR/IGF-IR hybrids [43]. Indeed, while the cell line we used was shown to contain IR protein by Western blotting [21], we have not been able to demonstrate any high affinity binding of ^125^I-insulin (Klaproth, B., and Sajid, W., unpublished data), suggesting that most of the IRs are drawn into hybrids which are unresponsive to insulin [43] but bind S961 [18] and IGF-I with high affinity. Also, we showed that the insulin-induced mitogenicity in these cells is not affected by siRNAs against the IR but only by siRNAs against the IGF-IR [44], suggesting that the insulin response is entirely through the IGF-IR. Since S961 binds poorly to the IGF-IR and there are no high-affinity IRs, the response must be through the hybrid receptors for which S961 has a high affinity. Finally, we show that the dose-response curve of S961-induced IR and Akt phosphorylation exactly matched the dose-response of S961-induced mitogenic effect. Therefore, taken together, we believe that our data strongly supported that the mixed agonist/antagonist activity of S961 was exerted through the IR and/or IR/IGF-IR hybrids. A hybrid receptor-mediated response may explain the fact that S961′s agonistic response shows a similar potency in cells that express mostly IRs (L6-hIR cells) or IGF-IRs (MCF-7 cells).

S961 has recently been used in rats as an IR antagonist, to block metabolism as well as mitogenic effects of the IR [45], [46]. We found that in the 1–10 nM range, S961 can in fact act as an agonist of IR-mediated mitogenic responses. Even though we did not find any agonistic effects of S961 on glycogen synthesis in differentiated preadipocytes or in L6-hIR cells as well as on lipogenesis in rat adipocytes, it cannot be ruled out that S961 could have agonistic effects in other cell types. Thus, our findings suggest that when using S961 as an IR antagonist *in vitro*, S961 concentrations well above 10 nM should be employed.

To our knowledge, together with the B29-B’29 crosslinked dimer, S961 is a rare example of mixed agonism/antagonism at the IR. Another peptide, S597 (a site 1-site 2 peptide), was previously shown to be a full agonist with respect to glycogen synthesis, but a partial agonist on cell proliferation in the presence of HI [17]. The 43 [18] and 31 [17] amino acids long peptides, S961 and S597, have structural similarities since they both consist of a site 1 and site 2 peptide although linked in different orders. None of the peptides show sequence similarity with HI although they were found to bind to the same IR binding sites as HI. The difference between the two peptides could be due to the orientation of the site 1 and site 2 peptides [47].

**Figure 6:**
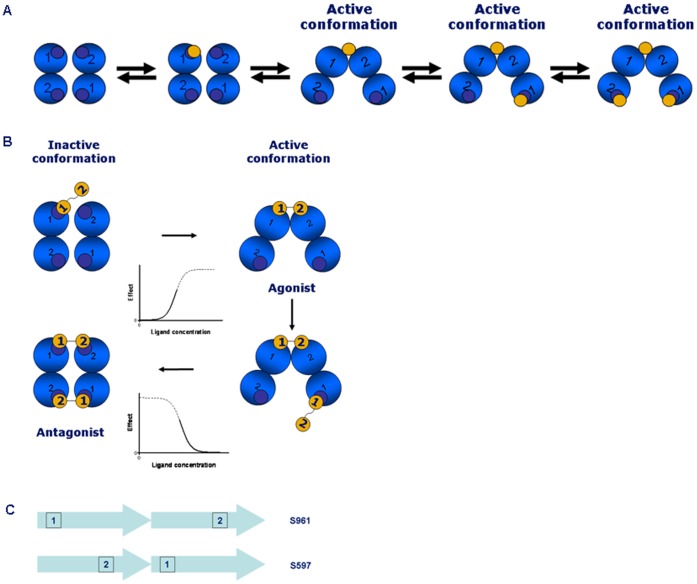
Current model of IR activation and proposed binding mechanism for S961. A . Current model of IR activation. The four blue circles represent the receptor binding sites (sites 1 and 2) seen from a top view. Insulin is depicted as a yellow circle. For a detailed explanation of binding sites 1 and 2, see [24]. **B**. Proposed binding mechanism for S961. The four blue circles represent the receptor binding sites (sites 1 and 2) seen from a top view. For a detailed explanation of binding sites 1 and 2, see [24]. The S961 peptide (Site 1–2 peptide) is shown as two connected yellow circles. At concentrations of 1–10 nM, S961 crosslinks the receptor, leading to agonist activity. At concentrations of above 10 nM, the higher flexibility of S961 in comparison to the insulin molecule allows simultaneous crosslinking of both pairs of binding sites, leading to an inactive conformation and antagonism. The corresponding activation and inactivation sigmoids are also shown. **C**. Orientation of peptide binding sites. If site 1 is located N-terminally and site 2 C-terminally, a longer distance between the binding sites in S961 in comparison to S661 can be achieved.

It is not established how the mixed agonist/antagonist properties of S961 arise. A plausible mechanism can be proposed based on the data presented in our study, and the current model of IR activation [12] which is schematically depicted in Fig. 6A. In this model, the IR molecule has two identical pairs (termed crosslinks) of partial sites (site 1 and site 2) arranged in an anti-parallel way. Insulin can bind first to any of the four available partial sites and then bind to the second site of the same crosslink (see Fig. 6A). It is believed that the crosslinked state of the receptor (with insulin bound to both partial sites) corresponds to the activated state of the receptor [8], [10].

The simplest model that can explain mathematically the bell shaped dose response of S961 is a two-site binding model, in which binding to one site activates the receptor and to the second site of lower affinity – inactivates it. Since IR has two identical pairs of partial sites, it is plausible to suggest that binding of the S961 peptide to the first pair of partial sites activates the receptor in a similar way as insulin does (see Fig. 6B). It is known that a second insulin molecule cannot bind simultaneously to the two partial sites of the second pair. However, it is hypothesised that the S961 peptide due to its flexibility can bind simultaneously to the two partial sites, albeit with a lower affinity. The second crosslinking event is postulated to result in the receptor inactivation, which might be a result of formation of a symmetrical “non-tilted” conformation of the receptor subunits (see Fig. 6B). In order to explain why S597 (site 2–1 peptide) is an agonist, whereas S961 (site 1–2 peptide) - agonist/antagonist, we suggest that S597 may not be capable of crosslinking the second pair partial sites and thus inactive the receptor as S961 does. We note that the distance between the actual receptor binding sites in these two peptides can be very different. If the receptor binding site in the site 1 peptide is positioned close to the N-terminus, and the receptor binding site of the site 2 peptide – close to the C-terminus, then a long distance between the receptor binding sites can be expected for the 1–2 peptide order (in the extended conformation of the peptide) as in S961, and a much shorter distance for the 2–1 peptide order as in S597 (see Fig. 6C). Thus, for the receptor binding sites positioned in S597 and S961 as in Fig. 6C, it is possible that the distance between the receptor binding sites in S961 is long enough for it to be capable of binding to the second crosslink and inactivate the receptor (Fig. 6C), but when the peptide order is reversed as in S597, the much shorter distance between the receptor binding sites (Fig. 6C) in S597 might prevent it from binding to the second crosslink. The proposed model is speculative, but consistent with the current knowledge of how insulin binds to the receptor [47]–[51]. Whether or not it is true requires further investigation and a better knowledge of the structure of the liganded receptor.

In summary, our results provide additional knowledge to the IR activation mechanism since we show that agonism and antagonism exist at IR. In addition, we provide in vitro studies which show that at 1 nM and 10 nM S961 can activate the IR and downstream signalling. Further exploration of the properties of such peptides should shed new light on the mechanism of IR activation and differential signalling.

## Supporting Information

Figure S1 **S661 antagonize IR and AKT phosphorylation in L6-hIR cells.** Cells were incubated in 12-wells plates with a cell density of 125,000 cells/well for three days, where after the cells were stimulated with increasing concentrations of S661 (panel **A** and **B**) or HI (panel **C** and **D**) in the presence of 3 nM HI or 10 µM S661, respectively. IR (pY1158) tyrosine phosphorylation (panel **A** and **C**) as well as AKT (pS473) (panel **B** and **D**) was measured. Data points represent average of three experiments. Error bars show one standard deviation.
(TIF)

## References

[1] Kasuga M, Karlsson FA, Kahn CR (1982) Insulin stimulates the phosphorylation of the 95,000-dalton subunit of its own receptor. Science 215, 185–187. 10.1126/science.7031900 7031900

[2] Petruzzelli LM, Ganguly S, Smith CJ, Cobb MH, Rubin CS, et al.. (1982) Insulin activates a tyrosine-specific protein kinase in extracts of 3T3-L1 adipocytes and human placenta. Proc. Natl. Acad. Sci. U. S. A. 79, 6792–6796. 10.1073/pnas.79.22.6792 PMC347219 6294652

[3] Ebina Y, Ellis L, Jarnagin K, Edery M, Graf L, et al.. (1985) The human insulin receptor cDNA: the structural basis for hormone-activated transmembrane signalling. Cell 40, 747–758. 10.1016/0092-8674(85)90334-4 2859121

[4] Ullrich A, Bell JR, Chen EY, Herrera R, Petruzzelli LM, et al.. (1985) Human insulin receptor and its relationship to the tyrosine kinase family of oncogenes. Nature 313, 756–761. 10.1038/313756a0 2983222

[5] Ullrich A, Gray A, Tam AW, Yang-Feng T, Tsubokawa M, et al.. (1986) Insulin-like growth factor I receptor primary structure: comparison with insulin receptor suggests structural determinants that define functional specificity. EMBO J. 5, 2503–2512. 10.1002/j.1460-2075.1986.tb04528.x PMC1167146 2877871

[6] Whittaker J, Okamoto AK, Thys R (1987) High-level expression of human insulin receptor cDNA in mouse NIH 3T3 cells. Proc. Natl. Acad. Sci. U. S. A. 84, 5237–5241. 10.1073/pnas.84.15.5237 PMC298830 3299382

[7] Hubbard SR, Till JH (2000) Protein tyrosine kinase structure and function. Annu. Rev. Biochem. 69, 373–398. 10.1146/annurev.biochem.69.1.373 10966463

[8] De Meyts P (1994) The structural basis of insulin and insulin-like growth factor-I receptor binding and negative co-operativity, and its relevance to mitogenic versus metabolic signalling. Diabetologia 37 Suppl 2, S135-S148. 10.1007/BF00400837 7821729

[9] De Meyts P, Roth J, Neville DM, Gavin JR, Lesniak MA (1973) Insulin interactions with its receptors: experimental evidence for negative cooperativity. Biochem. Biophys. Res. Commun. 55, 154–161. 10.1016/s0006-291x(73)80072-5 4361269

[10] Schäffer L (1994) A model for insulin binding to the insulin receptor. Eur. J. Biochem. 221, 1127–1132. 10.1111/j.1432-1033.1994.tb18833.x 8181471

[11] McKern NM, Lawrence MC, Streltsov VA, Lou MZ, Adams TE, et al.. (2006) Structure of the insulin receptor ectodomain reveals a folded-over conformation. Nature 443, 218–221. 10.1038/nature05106 16957736

[12] Kiselyov VV, Versteyhe S, Gauguin L, De Meyts P (2009) Harmonic oscillator model of the insulin and IGF1 receptors’ allosteric binding and activation. Mol. Sys. Biol 5, 243. 10.1038/msb.2008.78 PMC2657531 19225456

[13] Knudsen L, De Meyts, P, Kiselyov VV (2011) Insight into the molecular basis for the kinetic differences between the two insulin receptor isoforms. Biochem. J. 440(3), 397–403. 10.1042/BJ20110550 21838706

[14] Weiland M, Brandenburg C, Brandenburg D, Joost HG (1990) Antagonistic effects of a covalently dimerized insulin derivative on insulin receptors in 3T3-L1 adipocytes. Proc. Natl. Acad. Sci. U. S. A. 87, 1154–1158. 10.1073/pnas.87.3.1154 PMC53429 2153971

[15] Pillutla RC, Hsiao KC, Beasley JR, Brandt J, Ostergaard S, et al.. (2002) Peptides identify the critical hotspots involved in the biological activation of the insulin receptor. J. Biol. Chem. 277, 22590–22594. 10.1074/jbc.M202119200 11964401

[16] Schäffer L, Brissette RE, Spetzler JC, Pillutla RC, Ïstergaard S, et al.. (2003) Assembly of high-affinity insulin receptor agonists and antagonists from peptide building blocks. Proc. Natl. Acad. Sci. U. S. A. 100, 4435–4439. 10.1073/pnas.0830026100 PMC153573 12684539

[17] Jensen M, Hansen B, De Meyts P, Schäffer L, Ursø B (2007) Activation of the insulin receptor by insulin and a synthetic peptide leads to divergent metabolic and mitogenic signaling and responses. J. Biol. Chem. 282, 35179–35186. 10.1074/jbc.M704599200 17925406

[18] Schäffer L, Brand CL, Hansen BF, Ribel U, Shaw AC, et al.. (2008) A novel high-affinity peptide antagonist to the insulin receptor. Biochem. Biophys. Res. Commun. 376, 380–383. 10.1016/j.bbrc.2008.08.151 18782558

[19] Bonnesen C, Nelander G-M, Hansen BF, Jensen P, Krabbe JS, et al.. (2010) Synchronization in G0/G1 enhances the mitogenic response of cells overexpressing the human insulin receptor A isoform to insulin. Cell Biol. Toxicol. 26, 293–307. 10.1007/s10565-009-9142-x PMC2896650 19898946

[20] Listov-Saabye N, Jensen MB, Kiehr B, Hansen EW, Svendsen JE, et al.. (2009) MCF-7 human mammary adenocarcinoma cells exhibit augmented responses to human insulin on a collagen IV surface. J. Appl. Toxicol. 29, 470–477. 10.1002/jat.1428 19338014

[21] Oleksiewicz MB, Bonnesen C, Hegelund AC, Lundby A, Holm GM, et al.. (2011) Comparison of intracellular signalling by insulin and the hypermitogenic AspB10 analogue in MCF-7 breast adenocarcinoma cells. J. Appl. Toxicol. 31, 329–341. 10.1002/jat.1590 20936651

[22] Blagoev B, Kratchmarova I, Nielsen MM, Fernandez MM, Voldby J, et al.. (2002) Inhibition of Adipocyte Differentiation by Resistin-like Molecule α. J. Biol. Chem. 277, 42011–42016. 10.1074/jbc.M206975200 12189153

[23] Hansen BF, Danielsen GM, Drejer K, Sørensen AR, Wiberg FC, et al.. (1996) Sustained signalling from the insulin receptor after stimulation with insulin analogues exhibiting increased mitogenic potency. Biochem. J. 315, 271–279. 10.1042/bj3150271 PMC1217182 8670118

[24] Whitesell RR, Gliemann J (1979) Kinetic parameters of transport of 3-O-Methylglucose and glucose in adipocytes. J. Biol. Chem. 254, 5276–5283. 447648

[25] Taniguchi CM, Emanuelli B, Kahn CR (2006) Critical nodes in signalling pathways: insights into insulin action. Nat. Rev. Mol. Cell Biol. 7, 85–96. 10.1038/nrm1837 16493415

[26] Baker JG, Hill SJ (2007) Multiple GPCR conformations and signalling pathways: implications for antagonist affinity estimates. Trends Pharmacol. Sci. 28, 374–381. 10.1016/j.tips.2007.06.011 PMC2169386 17629959

[27] Fuh G, Cunningham BC, Fukunaga R, Nagata S, Goeddel DV, et al.. (1992) Rational design of potent antagonists to the human growth hormone receptor. Science 256, 1677–1680. 10.1126/science.256.5064.1677 1535167

[28] Ilondo MM, Damholt AB, Cunningham BA, Wells JA, De Meyts P, et al.. (1994) Receptor dimerization determines the effects of growth hormone in primary rat adipocytes and cultured human IM-9 lymphocytes. Endocrinology 134, 2397–2403. 10.1210/endo.134.6.8194466 8194466

[29] De Meyts P, Whittaker J (2002) Structural biology of insulin and IGF1 receptors: Implications for drug design. Nat. Rev. Drug Discov. 1, 769–783. 10.1038/nrd917 12360255

[30] Levitzki A, Mishani E (2006) Tyrphostins and other tyrosine kinase inhibitors. Annu. Rev. Biochem. 75, 93–109. 10.1146/annurev.biochem.75.103004.142657 16756486

[31] Leahy DJ (2008) A molecular view of anti-ErbB monoclonal antibody therapy. Cancer Cell 13, 291–293. 10.1016/j.ccr.2008.03.010 18394550

[32] Tager H, Thomas N, Assoian R, Rubenstein A, Saekow M, et al.. (1980) Semisynthesis and biological activity of porcine [LeuB24]insulin and [LeuB25]insulin. Proc. Natl. Acad. Sci. U. S. A. 77, 3181–3185. 10.1073/pnas.77.6.3181 PMC349578 6997872

[33] Olefsky JM, Saekow M, Tager H, Rubenstein AH (1980) Characterization of a mutant human insulin species. J. Biol. Chem. 255, 6098–6105. 6993466

[34] Kobayashi M, Ohgaku S, Iwasaki M, Maegawa H, Shigeta Y, et al.. (1982) Characterization of [LeuB-24]- and [LeuB-25]-insulin analogues. Receptor binding and biological activity. Biochem. J. 206, 597–603. 10.1042/bj2060597 PMC1158628 6756393

[35] Diaconescu C, Saunders D, Gattner HG, Brandenburg D (1982) [Leu(B24)]- and [Leu(B25)]insulins are not antagonists of lipogenesis in adipocytes. Hoppe-Seylers’s Z. Physiol. Chem. 363, 187–192. 10.1515/bchm2.1982.363.1.187 7037593

[36] Keefer LM, Piron MA, De Meyts P, Gattner HG, Diaconescu C, et al.. (1981) Impaired negative cooperativity of the semisynthetic analogues human [LeuB24]- and [LeuB25]-insulins. Biochem. Biophys. Res. Commun. 100, 1229–1236. 10.1016/0006-291x(81)91955-0 7023479

[37] Figlewicz DP, Best JD, Tager HS, Taborsky GJ Jr (1983) LeuB24]insulin is an insulin agonist at the liver in vivo. Am. J. Physiol. 245, E483–E488. 10.1152/ajpendo.1983.245.5.E483 6356934

[38] De Meyts P, Van Obberghen E, Roth J, Brandenburg D, Wollmer A (1978) Mapping of the residues responsible for the negative cooperativity of the receptor-binding region of insulin. Nature 273, 504–509. 10.1038/273504a0 661960

[39] Soos MA, Siddle K, Baron MD, Heward JM, Luzio JP, et al.. (1986) Monoclonal antibodies reacting with multiple epitopes on the human insulin receptor. Biochem. J. 235, 199–208. 10.1042/bj2350199 PMC1146668 2427071

[40] Soos MA, Field CE, Lammers R, Ullrich A, Zhang B, et al.. (1992) A panel of monoclonal antibodies for the type I insulin-like growth factor receptor. Epitope mapping, effects on ligand binding, and biological activity. J. Biol. Chem. 267, 12955–12963. 1377676

[41] Gu JL, Goldfine ID, Forsayeth JR, De Meyts P (1988) Reversal of insulin-induced negative cooperativity by monoclonal antibodies that stabilize the slowly dissociating (“Ksuper”) state of the insulin receptor. Biochem. Biophys. Res. Commun. 150, 694–701. 10.1016/0006-291x(88)90447-0 3277631

[42] Verspohl EJ, Maddux BA, Goldfine ID (1988) Insulin and insulin-like growth factor I regulate the same biological functions in HEP-G2 cells via their own specific receptors. J. Clin. Endocrinol. Metab. 67, 169–174. 10.1210/jcem-67-1-169 2837499

[43] Benyoucef S, Surinya KH, Hadaschik D, Siddle K (2007) Characterization of insulin/IGF hybrid receptors: Contributions of the insulin receptor L2 and Fn1 domains and the alternatively spliced exon 11 sequence to ligand binding and receptor activation. Biochem. J., 403, 603–613. 10.1042/BJ20061709 PMC1876384 17291192

[45] Vikram A, Kushwaha S, Jena GB (2011) Relative influence of testosterone and insulin in the regulation of prostatic cell proliferation and growth. Steroids 76, 416–423. 10.1016/j.steroids.2010.12.014 21215763

[46] Vikram A, Jena G (2010) S961, an insulin receptor antagonist causes hyperinsulinemia, insulin-resistance and depletion of energy stores in rats. Biochem. Biophys. Res. Commun. 398, 260–265. 10.1016/j.bbrc.2010.06.070 20599729

[47] Ward CW, Lawrence MC (2009) Ligand-induced activation of the insulin receptor: a multi-step process involving structural changes in both the ligand and the receptor. BioEssays 31, 422–434. 10.1002/bies.200800210 19274663

[48] Whittaker J, Whittaker LJ, Roberts CT Jr, Phillips NB, Ismail-Beigi F, et al.. (2012) α-Helical element at the hormone-binding surface of the insulin receptor functions as a signalling element to activate its tyrosine kinase. Proc. Natl. Acad. Sci. U.S.A. 109, 11166–11171. 10.1073/pnas.1205681109 PMC3396503 22736795

[49] Menting JG, Ward CW, Margetts MB, Lawrence MC (2009) A thermodynamic study of ligand binding to the first three domains of the human insulin receptor: Relationship between the receptor α-chain c-terminal peptide and the site 1 insulin mimetic peptides. Biochemistry 48, 5492–5500. 10.1021/bi900261q 19459609

[50] Smith BJ, Huang K, Kong G, Chan SJ, Nakagawa S, et al.. (2010) Structural resolution of a tandem hormone-binding element in the insulin receptor and its implications for design of peptide agonists. Proc. Natl. Acad. Sci. U.S.A. 107, 6771–6776. 10.1073/pnas.1001813107 PMC2872410 20348418

[51] Ward CW, Lawrence MC (2012) Similar but different: ligand-induced activation of the insulin and epidermal growth factor receptor families. Curr. Opin. Struct. Biol. 22, 360–366. 10.1016/j.sbi.2012.03.014 22521506

