# Supplementary figures and images for: Agonism and Antagonism at the Insulin Receptor
Source: PLoS One. 2012 Dec 27;7(12):e51972. doi: 10.1371/journal.pone.0051972 (PMC3531387; doi:10.1371/journal.pone.0051972)

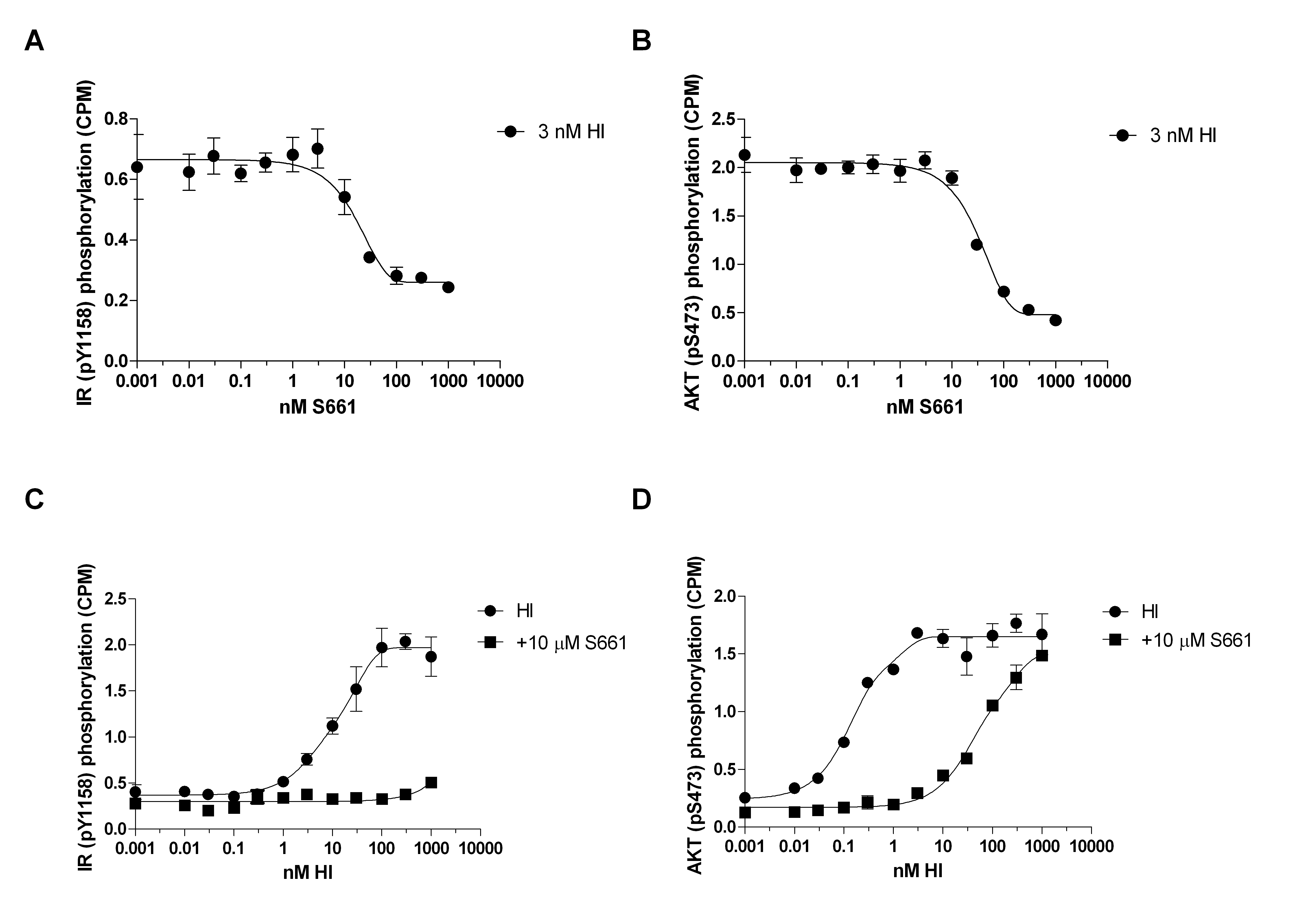

Supplement: Figure S1 — S661 antagonize IR and AKT phosphorylation in L6-hIR cells. Cells were incubated in 12-wells plates with a cell density of 125,000 cells/well for three days, where after the cells were stimulated with increasing concentrations of S661 (panel A and B) or HI (panel C and D) in the presence of 3 nM HI or 10 µM S661, respectively. IR (pY1158) tyrosine phosphorylation (panel A and C) as well as AKT (pS473) (panel B and D) was measured. Data points represent average of three experiments. Error bars show one standard deviation. (TIF) [file pone.0051972.s001.tif]
